# Utilizing an artificial intelligence framework (conditional generative adversarial network) to enhance telemedicine strategies for cancer pain management

**DOI:** 10.1186/s44158-023-00104-8

**Published:** 2023-06-20

**Authors:** Marco Cascella, Giuliana Scarpati, Elena Giovanna Bignami, Arturo Cuomo, Alessandro Vittori, Piergiacomo Di Gennaro, Anna Crispo, Sergio Coluccia

**Affiliations:** 1 Department of Anesthesia and Critical Care, Istituto Nazionale Tumori-IRCCS, Fondazione Pascale, 80100 Naples, Italy; 2 Department of Medicine, Surgery and Dentistry “Scuola Medica Salernitana, ” University of Salerno, 84084 Baronissi, SA Italy; 3 Critical Care and Pain Medicine Division, Department of Medicine and Surgery, University of Parma, Viale Gramsci 14, 43126 Parma, Italy; 4 Department of Anesthesia and Critical Care, ARCO Roma, Ospedale Pediatrico Bambino Gesù IRCCS, Rome, Italy; 5 Epidemiology and Biostatistics Unit, Istituto Nazionale Tumori-IRCCS, Fondazione Pascale, 80100 Naples, Italy

**Keywords:** Telemedicine, Cancer pain, Artificial intelligence, Machine learning, Conditional generative adversarial network, Deep learning

## Abstract

**Background:**

The utilization of artificial intelligence (AI) in healthcare has significant potential to revolutionize the delivery of medical services, particularly in the field of telemedicine. In this article, we investigate the capabilities of a specific deep learning model, a generative adversarial network (GAN), and explore its potential for enhancing the telemedicine approach to cancer pain management.

**Materials and methods:**

We implemented a structured dataset comprising demographic and clinical variables from 226 patients and 489 telemedicine visits for cancer pain management. The deep learning model, specifically a conditional GAN, was employed to generate synthetic samples that closely resemble real individuals in terms of their characteristics. Subsequently, four machine learning (ML) algorithms were used to assess the variables associated with a higher number of remote visits.

**Results:**

The generated dataset exhibits a distribution comparable to the reference dataset for all considered variables, including age, number of visits, tumor type, performance status, characteristics of metastasis, opioid dosage, and type of pain. Among the algorithms tested, random forest demonstrated the highest performance in predicting a higher number of remote visits, achieving an accuracy of 0.8 on the test data. The simulations based on ML indicated that individuals who are younger than 45 years old, and those experiencing breakthrough cancer pain, may require an increased number of telemedicine-based clinical evaluations.

**Conclusion:**

As the advancement of healthcare processes relies on scientific evidence, AI techniques such as GANs can play a vital role in bridging knowledge gaps and accelerating the integration of telemedicine into clinical practice. Nonetheless, it is crucial to carefully address the limitations of these approaches.

## Introduction

The utilization of telemedicine is progressively rising in various medical domains, transforming the way healthcare is delivered [1]. This technology-driven approach allows for remote patient care, enabling healthcare professionals to provide medical services, including pain management, through virtual platforms [2, 3]. Within the realms of oncology and pain medicine, telemedicine has emerged as a promising tool for addressing the complex needs of patients experiencing cancer-related pain [4]. It offers, indeed, a convenient and accessible means of delivering healthcare services, especially for patients who may face challenges in accessing specialized care due to geographic distance, physical limitations, or other impediments. Remarkably, patients can receive expert care without the need for frequent, time-consuming visits to healthcare facilities, reducing the burden of travel and associated costs. Moreover, telemedicine allows for increased flexibility in scheduling appointments, making it easier for patients to receive timely care and support [5].

However, telemedicine for cancer-related pain management also entails different challenges [6]. General issues such as security and privacy of patient data, and technical problems, are common to telemedicine applications in various medical fields. Nevertheless, it is crucial to establish the dynamics of the care process to adapt the functionality of the system to meet the clinical needs of patients suffering from cancer pain. Within the framework of personalized care, it is necessary to establish calibrated and dynamic pathways that can anticipate the need for in-person visits, the requirement for further diagnostic investigations, and the appropriate timing of clinical reassessments, for example, for opioid titration [7].

The integration of artificial intelligence (AI) in healthcare has enormous potential to transform the provision of medical services. AI algorithms have the capability to uncover patterns, correlations, and trends within diverse datasets that may remain unnoticed by human observers. Consequently, by leveraging AI’s capabilities in data analysis, pattern recognition, and decision-making, healthcare professionals can enhance the efficiency and effectiveness of telemedicine strategies. Therefore, healthcare providers are empowered to execute more informed decisions and can customize treatment approaches to meet the specific needs of individual patients. Different AI and machine learning (ML) strategies have been previously evaluated by our research group for this purpose [8]. In this article, we explore the capabilities of a specific AI system, generative adversarial network (GAN), and its potential applications in the context of our research focus. GAN is an AI framework that consists of two antagonistic neural networks: the generator and the discriminator network. Notably, GANs play a notable role in machine learning (ML) by generating new data that closely resembles a given dataset. These techniques offer exciting prospects in medical research, revolutionizing various aspects of healthcare including medical imaging [9], data synthesis [10], disease diagnosis [11], and drug discovery [12].

In this article, we evaluate the potential applications of GANs for implementing a structured dataset and enhancing descriptive and predictive analyses.

## Material and methods

### Study population and model of care

The study population comprised adult patients receiving telemedicine-based treatment for cancer pain at the Istituto Nazionale Tumori, Fondazione Pascale, Italy.

To provide comprehensive care, a hybrid model was implemented. The initial phase involved an in-person visit where a thorough clinical and instrumental evaluation was conducted. During this visit, legal and regulatory concerns, including obtaining informed consent, collecting essential data, and delivering patient training, were also addressed. Subsequently, synchronous real-time video consultations were scheduled based on the patient’s clinical needs. Additional remote follow-up consultations were programmed or arranged as per the patients’ requirements. Face-to-face visits were also allowed for performing minimally invasive procedures, diagnostic purposes, addressing acute clinical concerns (e.g., managing drug side effects), or upon the patient’s request [5].

The study was approved by the local Medical Ethics Committee (protocol code 41/20 Oss; date of approval: 26 November 2020), and all participating patients provided written informed consent. The investigation adhered to the principles outlined in the Declaration of Helsinki.

### Dataset implementation

The dataset under analysis comprised 226 patients and a total of 489 remote visits following the local telemedicine program. These visits were conducted from March 2021 to September 2022.

The structured dataset included demographic variables, such as age and gender along with clinical data including cancer type, Eastern Cooperative Oncology Group Performance Status (ECOG-PS), opioid prescription as morphine equivalent dose (MED), the presence of metastasis (including bone metastases as a separate variable), number of remote consultations, and pro-capita teleconsultations. Additionally, process variables such as the dropout rate from the remote process, multiprofessional consultations, and the distribution of visits across the national territory were considered. Pain features included background pain (nociceptive, neuropathic) and breakthrough cancer pain (BTcP) [13].

### Generative adversarial network application

From the original dataset, the following variables were considered: age, gender, cancer type, metastases, bone metastases, BTcP status and type (neuropathic), MED, and the number of teleconsultations. A cleaning procedure was performed on data to encode categorical variables and to standardize numeric ones. Data were split in training and testing set in a 70–30% ratio. The deep learning model, namely conditional GAN, was employed to generate synthetic samples that closely resemble the attributes and patterns observed in the real data. This AI framework consists of a tandem pair of deep neural networks (DNNs): G as the generator and D as the discriminator. The generator, G, learns the training data distribution and generates observations accordingly, while the discriminator, D, assesses the likelihood of a sample originating from the training data or from G. Consequently, the objective of G is to optimize the probability of D making an error. D functions as a binary classifier, distinguishing between real data and the generated data from G [14].

**Fig. 1:**
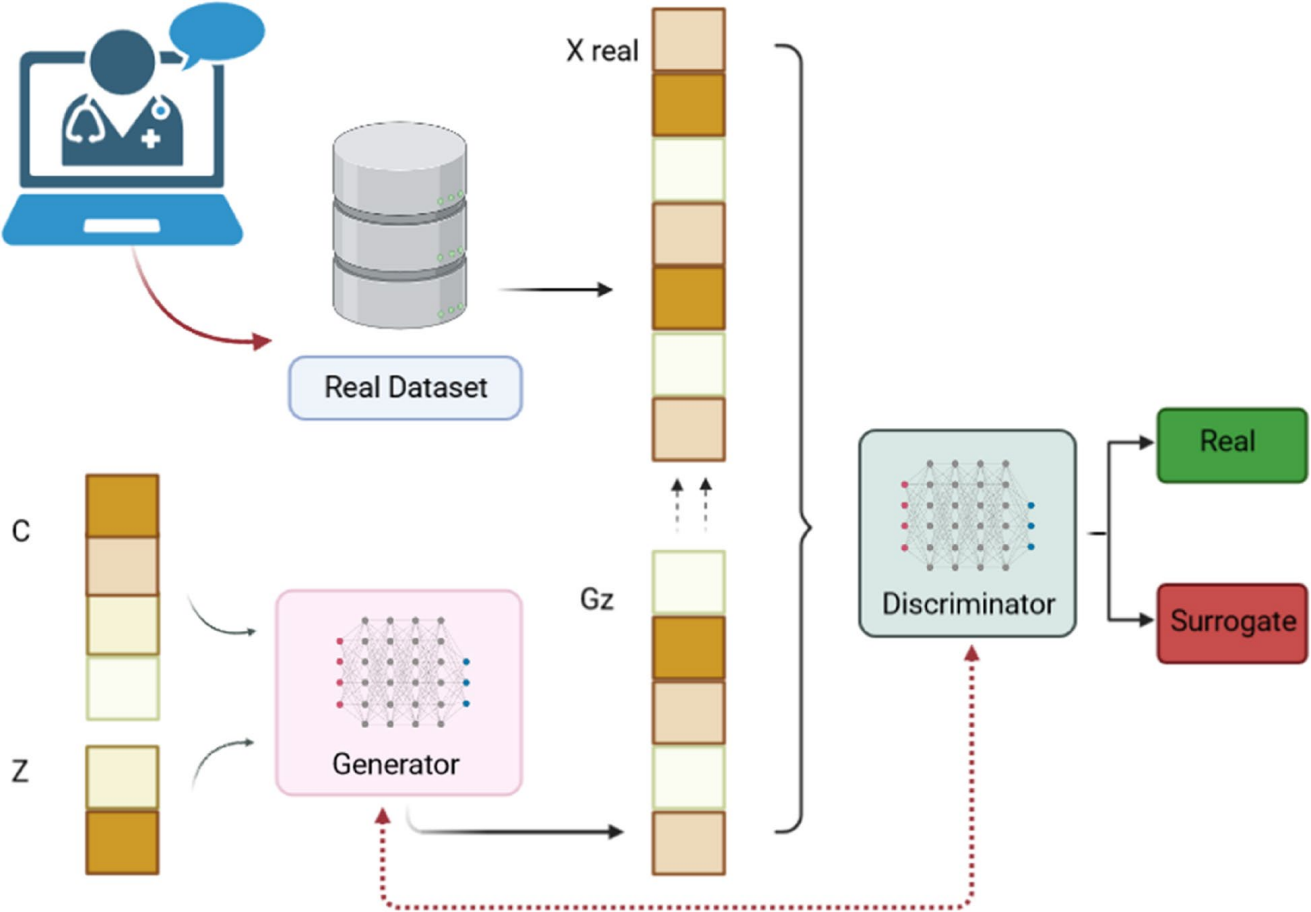
In a conditional generative adversarial network (cGAN), both the generator (G) and the discriminator (D) are influenced by additional information provided as an extra input. In the context of a GAN, a noise vector (*z*) refers to a random input that is fed into the G model; it acts as a source of randomness, providing variation and unpredictability to the generated samples. Consequently, the G output is denoted as G(z). By manipulating the noise vector, the G can produce different outputs, allowing for the generation of diverse and unique samples. The class label (c) refers to the predefined category or class to which a data sample belongs. It is an essential component in supervised learning tasks, where the goal is to train a model to predict the class of unseen data based on labeled training examples. Each data sample is associated with a specific class label, indicating its category or group membership. Through the conditioning information, the cGAN can generate more targeted and contextually relevant outputs (prediction labels), enhancing its ability to produce desired results based on the provided extra information

In simple terms, the generator’s goal is to produce data that closely resembles real data, to the point of being indistinguishable. Initially, the G output may be random, but through training, it learns to generate data that simulates the real examples in the training dataset. On the other hand, the D’ role is to differentiate between the fake examples generated by G and real examples from the actual data domain. Its purpose is to classify input examples as either belonging to G or the real data distribution. In the case of a conditional GAN (cGAN), both G and D are conditioned to have knowledge about the specific type or category of data they are handling. This conditioning enables them to generate or discriminate samples based on the provided information, ensuring that the generated samples are coherent and aligned with the desired category or condition (Fig. 1).

The ability of D to distinguish between real and generated samples is calculated through a binary cross-entropy function [15]. The conditional probability is used for both the generator and the discriminator, instead of the regular one. Two neural networks (i.e., D and G) work in a minimax game:

$${\mathrm{min}}_{\mathrm{G}}{\mathrm{max}}_{\mathrm{D}}V\left(D,G\right)={E}_{{x\sim p}_{data}(x)}[\mathrm{log}D(x)]+{E}_{z\sim {p}_{z}\left(z\right)}\{\mathrm{log}[1-D(G\left(z\right))]\}$$

In the formula, V(D, G) represents the value function, namely the objective function used to train the D network and evaluate its performance in classifying real and generated samples; “*p*_z_” is the probability distribution of the latent space or the input noise vector; and “*p*_data_” refers to the distribution of the real data samples from the actual data domain and represents the probability distribution of the real data that the GAN is trying to learn and replicate [16]. Finally, the G output is denoted as G(z).

By optimizing the value function, the D network learns to become more accurate in distinguishing real data from generated data. This, in turn, guides the D network to improve its ability to generate samples that resemble the real data distribution, as it aims to fool D.

### Predictive analysis

The predictive analysis of the complete dataset focused on the variables associated with a higher number of remote visits.

#### Preprocessing and exploratory data analysis

After loading the generated dataset, a series of preparation processes or preprocessing steps were conducted, including normalization and standardization. Numerical variables were standardized as categorical variables and encoded as the presence/absence of a single modality. Following this, exploratory data analysis was performed to uncover any discernible trends within the dataset. Based on the results of the exploratory analysis, the variables to be included in the subsequent analyses were carefully selected. Finally, a univariate analysis was performed on real data to establish the main associations between remote consultations (categorized as “one” and “more than one”) and main features.

#### Machine learning algorithms

Four machine learning (ML)-based algorithms were utilized in this study:

- 1. LASSO-RIDGE regression (elastic model): This algorithm is a generalized linear regression model that applies a penalty to the loss function by resizing the regressors. The majority of regressors are shrunk or set to zero if they are not deemed important in explaining the dependent variable. This approach effectively reduces model complexity and safeguards against overfitting [17].
- 2. Random forest (RF) algorithm: It is a versatile algorithm that can be used for both regression and classification tasks. The algorithm belongs to the category of bagging methods and is widely popular in ML. RF works by constructing multiple decision tree models and aggregating their outputs to improve overall performance [18].
- 3. Gradient boosting machine (GBM): GBM is designed to optimize predictions by iteratively improving on the errors of previous tree regression or classification models. It belongs to the boosting method category and aims to reduce the overall error function, allowing each subsequent model to build upon the strengths of its predecessors [19].
- 4. Single hidden layer artificial neural network (ANN): This approach involves a neural network architecture with a single hidden layer. It minimizes a loss function by adjusting weights that govern the connections between neurons in adjacent layers. ANN is commonly used for classification and regression problems [20].

These four ML-based models were chosen to implement different approaches for regression or classification tasks. RF and GBM were selected for their bagging and boosting techniques, respectively, to enhance predictive performance in regression models. LASSO-RIDGE was chosen as a binary regression model to compare different numerical approaches. ANN emerges as a potent learning algorithm extensively employed for classification (and regression) tasks.

#### Model processing and evaluation

Since the objective of this study was to predict the likelihood of cancer patients requiring multiple remote consultations, the outcome variable was “the number of remote consultations” dichotomized. Each classifier was optimized using repeated cross-validation techniques, which involved calculating the mean error through K-fold cross-validation and determining the hyperparameters that yield the best predictions and capture the underlying structure. The dataset was divided into a training set (80% of the total size) for hyperparameter identification and a test set (20% of the total size) for model evaluation.

To assess the performance of the models, various metrics were utilized, including accuracy and the area under the receiver operating characteristic (ROC) curve (AUC). The AUC represents the trade-off between sensitivity and 1-specificity. When the modalities of the outcome variable are equally distributed, the AUC closely approximates accuracy. However, it is also suitable for cases with imbalanced modality distribution. Each threshold provides a conditional correct classification rate, and the AUC quantifies the probability of a randomly selected positive response being ranked higher than a randomly selected negative response.

Another evaluation metric used is the F1 score. It considers both precision and recall. Precision is the ratio of true positives to the sum of true positives and false positives, while recall is the ratio of true positives to the sum of true positives and false negatives. The F1 score combines these two metrics and provides a balanced measure of the model’s performance. It ranges from 0 to 1, where a higher value indicates better predictive ability.

$$\mathrm{F}1=2\times (\mathrm{precision}\times \mathrm{recall})/(\mathrm{precision}+\mathrm{recall})$$

### Risk analysis

Based on the ML processes, a risk analysis was conducted to assess the potential increase in remote consultations (greater than one). We employed an odds-ratio-like analysis referred to as simulated odds ratios (SORs). Simulations were performed to evaluate the risk associated with a higher number of consultations in target individuals. Around 500 simulations were conducted 150 times to establish a classification rate for both the cases (target individuals) and control individuals. Subsequently, we calculated the odds ratio by comparing the effective odds for each individual type and determined the 95% credibility intervals (95% CIs) as the effective 2.5 and 97.5 percentiles for the SOR samples. While numerous possibilities were considered, we defined four standard clinical conditions (targets) as follows:

• Condition 1: Younger cancer patients (mean age of 45 years) versus older (mean age of 75 years)
• Condition 2: The presence or not of BTcP
• Condition 3: The presence or not of neuropathic pain

### Statistical analysis

Univariate comparisons as *t*-tests were made between real and fake data to experiment with such results. The number of tele-visits was chosen as the target feature and discretized in one tele-visit or > 1 tele-visits (“more”).

Main statistics were utilized to admit comparisons. Generation phase and comparison plots were performed using Python 3.8.0, and classifiers and their performances and univariable analyses were assessed using *R* 4.1.3 environment. The utilized libraries consisted of Python’s TabGAN and Seaborn, along with R’s caret and ggplot2.

## Results

### AI-derived dataset and analyses

Table 1 presents the results of the univariate analysis conducted on the data.

**Table 1:** Univariate analysis on generated and actual data

Variable	**Generated data**			**Real data**		
	**One** *N* = 112^1^	**More***N* = 106^1^	***p*-value**^2^	**One** *N* = 108^1^	**More** *N* = 110^1^	***p*-value**^2^
**Age (years)**			0.056			**0.013**
*N*	112	106		108	110	
*Mean (SD)*	66 (12)	63 (10)		66 (12)	62 (12)	
*Median (IQR)*	66 (57, 76)	63 (54, 70)		67 (58, 76)	63 (53, 71)	
**Gender**			0.058			**0.043**
*Female*	49 (44%)	60 (57%)		49 (45%)	65 (59%)	
*Male*	63 (56%)	46 (43%)		59 (55%)	45 (41%)	
**ECOG**			0.063			0.324
*N*	112	106		108	110	
*Mean (SD)*	2 (1)	3 (1)		2 (1)	2 (1)	
*Median (IQR)*	2 (2, 3)	2 (2, 3)		2 (2, 3)	2 (2, 3)	
**Cancer**						
*Bladder*	11 (9.8%)	8 (7.5%)		10 (9.3%)	6 (5.5%)	
*Breast*	14 (12%)	21 (20%)		10 (9.3%)	20 (18%)	
*Gastrointestinal*	31 (28%)	16 (15%)		26 (24%)	16 (15%)	
*Gynecological*	2 (1.8%)	3 (2.8%)		3 (2.8%)	6 (5.5%)	
*Head & neck*	4 (3.6%)	5 (4.7%)		6 (5.6%)	8 (7.3%)	
*Kidney*	0 (0%)	0 (0%)		1 (0.9%)	5 (4.5%)	
*Lung*	19 (17%)	15 (14%)		17 (16%)	14 (13%)	
*Melanoma/skin*	0 (0%)	0 (0%)		3 (2.8%)	2 (1.8%)	
*Pancreas*	4 (3.6%)	7 (6.6%)		2 (1.9%)	8 (7.3%)	
*Prostate*	10 (8.9%)	6 (5.7%)		10 (9.3%)	4 (3.6%)	
*Soft tissue & bones*	8 (7.1%)	12 (11%)		9 (8.3%)	13 (12%)	
*Other sites*	9 (8.0%)	13 (12%)		11 (10%)	8 (7.3%)	
**Bone metastases**			0.083			0.795
*No*	67 (60%)	51 (48%)		58 (54%)	61 (55%)	
*Yes*	45 (40%)	55 (52%)		50 (46%)	49 (45%)	
**BTcP**			0.115			0.551
*No*	78 (70%)	63 (59%)		70 (65%)	67 (61%)	
*Yes*	34 (30%)	43 (41%)		38 (35%)	43 (39%)	
**Neuropathic pain**			0.242			0.161
*No*	69 (62%)	57 (54%)		69 (64%)	60 (55%)	
*Yes*	43 (38%)	49 (46%)		39 (36%)	50 (45%)	
**MED**			0.063			0.805
≤ *60*	53 (47%)	37 (35%)		43 (40%)	42 (38%)	
> *60*	59 (53%)	69 (65%)		65 (60%)	68 (62%)	

Legend: ^1^*n* (%); ^2^Pearson’s chi-squared test; Wilcoxon rank-sum test. Abbreviations: *ECOG*, Eastern Cooperative Oncology Group; *BTcP*, breakthrough cancer pain; *MED*, morphine equivalent dose

In the cGAN-derived dataset, the model replicated the same number of “fake” cancer patients and generated 218 artificial samples [21]. In the original dataset (*n* = 226), 8 patients were excluded due to incomplete data.

**Fig. 2:**
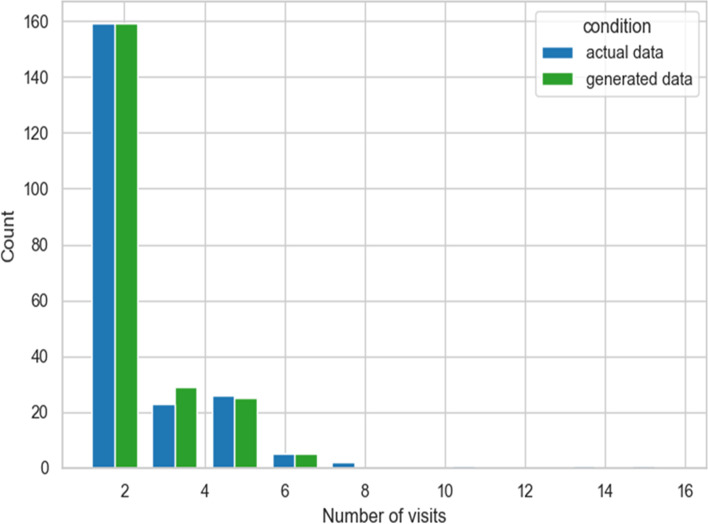
Number of remote consultations. Actual data (from the original dataset) and those generated by the implemented deep learning approach

The analysis of the obtained dataset showed that the number of remote consultations [mean (µ) 2.1; standard deviation (σ) 1.6; median 2) was similarly distributed by generated and real data (means 2.2 and 2, respectively, *p* = 0.24, see Fig. 2) and almost equally split in “one” and “more” tele-visits (50–50% in generated data, 49.5–50.5% in real data).

**Fig. 3:**
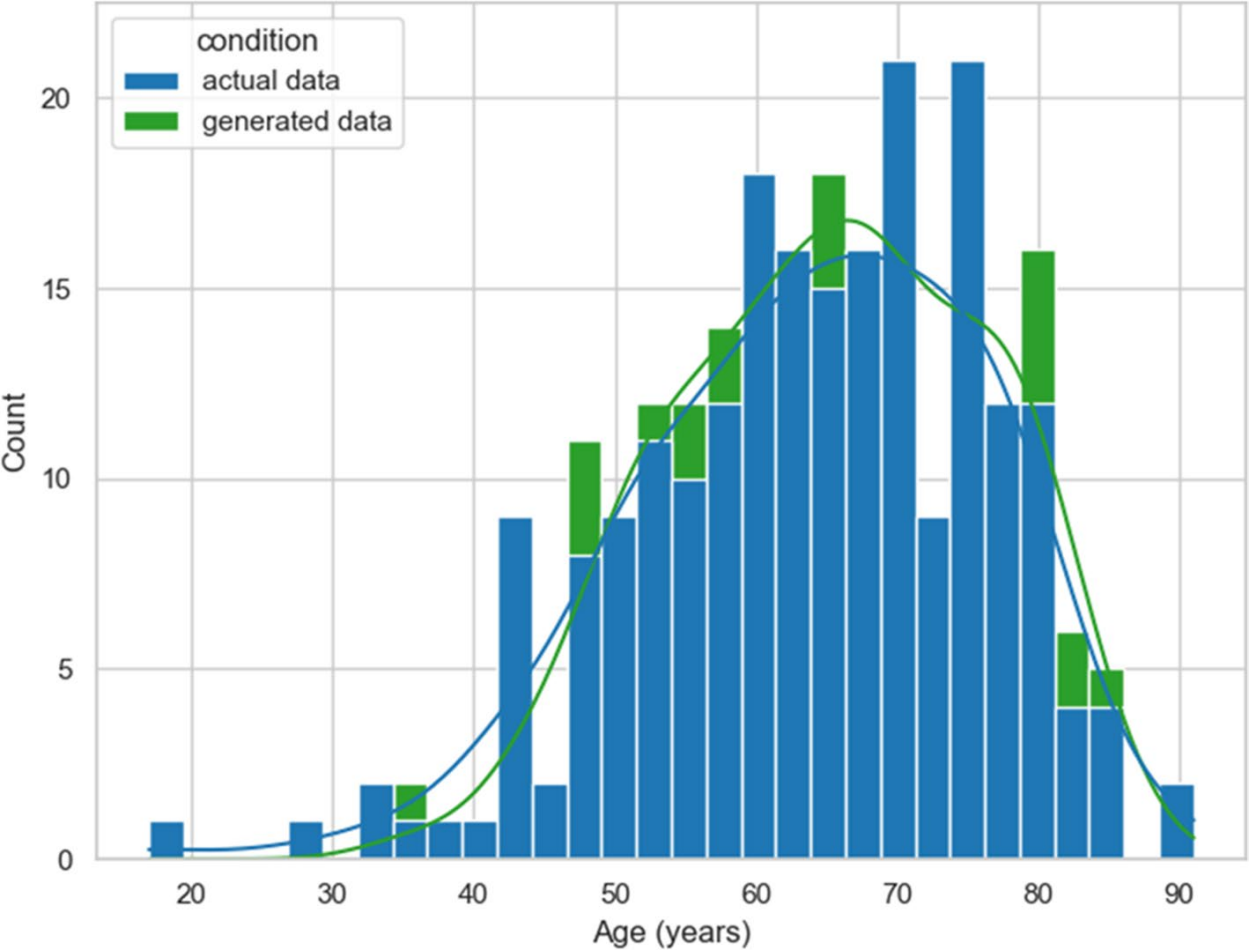
Patients’ age. Actual data (from the original dataset) and those generated by the implemented deep learning approach

The mean cancer patients’ age was 63.9 years (*σ* = 12.3) in real data, as not statistically different (*p* = 0.22) or lower (*p* = 0.11) from generated data (*µ* = 65.3, *σ* = 11.7). The probability of doing more than one tele-visit decreased by 3% for each year of age (*p* < 0.01); no differences were detected by type of data (Wald test *p* = 0.92) (Fig. 3).

**Fig. 4:**
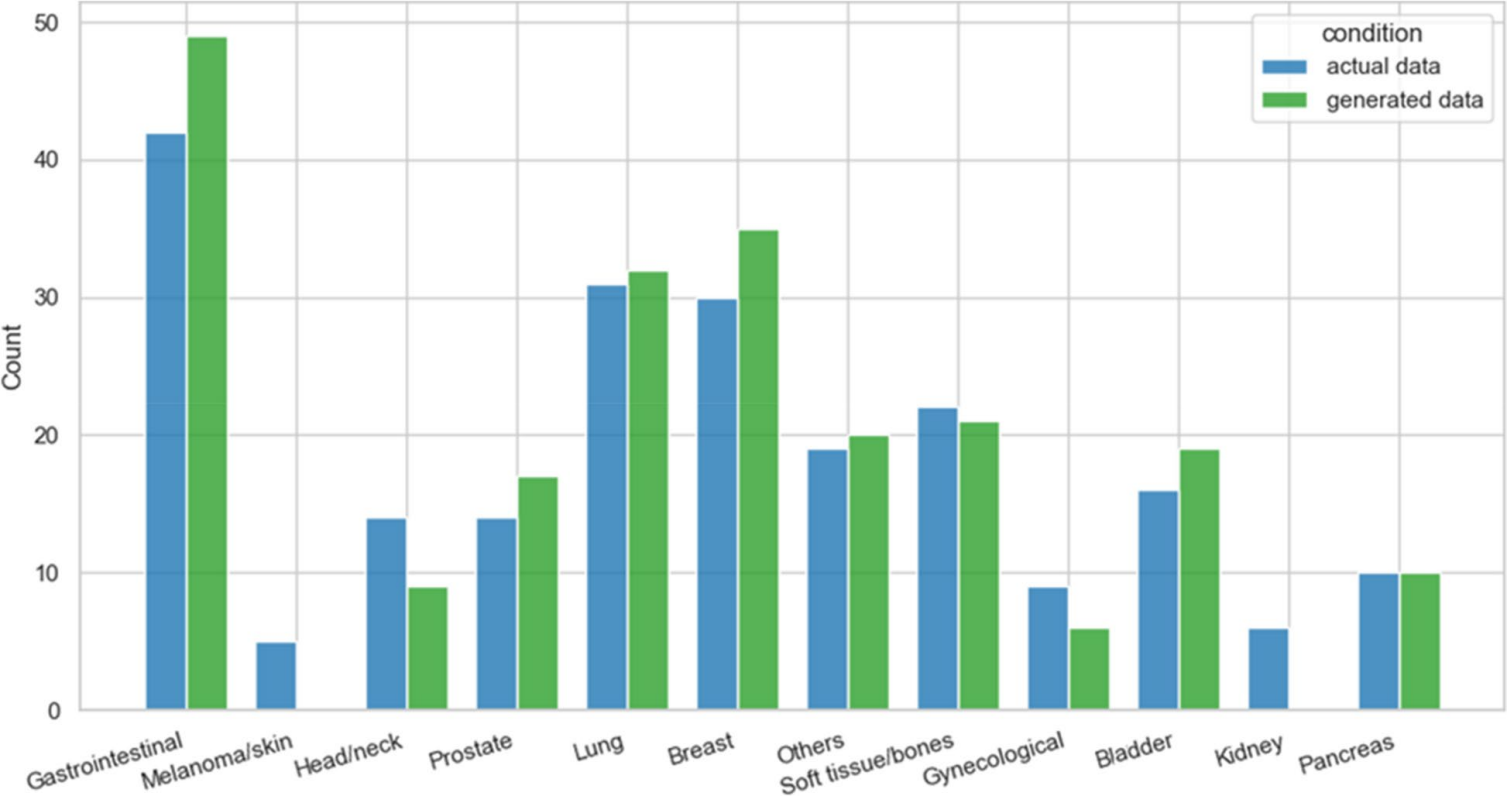
Tumor site. Actual data (from the original dataset) and those generated by the implemented deep learning approach

The site of the tumor was similarly reproduced. Melanoma and kidney cancers were less represented in the real data, and these were not replicated by cGAN (Fig. 4).

**Fig. 5:**
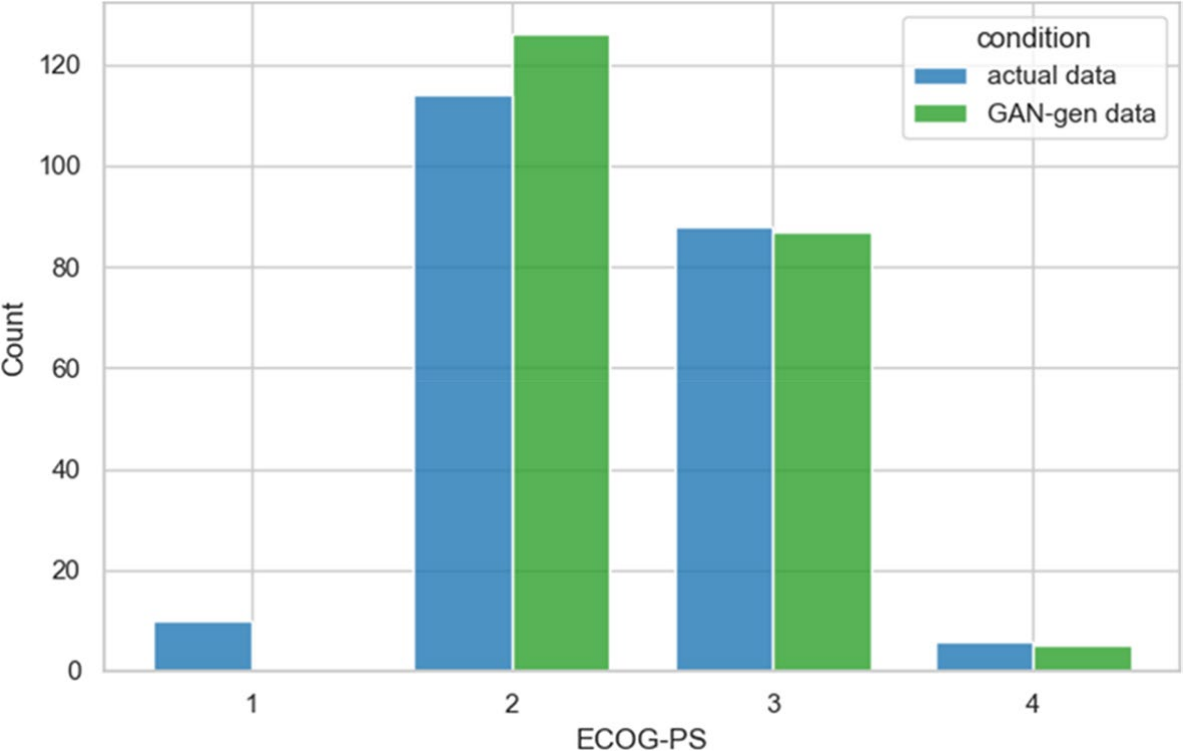
Eastern Cooperative Oncology Group Performance Status (ECOG-PS). Data from the original and the generated dataset

The ECOG-PS was similarly distributed by type of data (Wilcoxon rank-sum test *p* = 0.6; *µ* = 2.4 and *µ* = 2.5 for real and generated data, respectively; same medians = 2) (Fig. 5).

No differences were found for gender (*p* = 0.7), bone metastases (*p* = 1.0), MED (> 60, 61% for real data and 58.7% for generated data, *p* = 0.7), BTcP (*p* = 0.76, for real data 37.2% and 35.3% from the GAN), and neuropathic condition (40.8% for real data, 42.2% for generated data, *p* = 0.85).

### Selection of the optimal ML model

Subsequently, the four ML models were evaluated. As 90.6% of cancer patients (89.9% in real data, 91.3% in fake data) were metastatic, the presence of metastases was removed from the predictive features set.

The GBM classifier consisted of 100 trees with a maximum depth of 5 splits, a shrinkage parameter of 0.1, and minimum leaf observations set to 15. For the LASSO model, the mixing parameter (*α*) was set to 0 and the shrinkage (*λ*) to 0.25. The selected RF model utilized 13 randomly chosen variables for each tree. The best ANN configuration consisted of a hidden layer with 10 neurons and regularization parameters set to 0.035.

The RF model outperformed all other models, demonstrating an impressive accuracy of 0.8 on the test data. The performances of classifiers are described in Table 2.

**Table 2:** Performances of the considered machine learning algorithms. RF emerged as the top-performing model with an accuracy of 0.8 on the test data and a specificity of 0.71

Classifier	AUC	ACC (TR)	ACC (TST)	L (TST)	U (TST)	P	SENS (TST)	SPEC (TST)	F1
GBM	0.87	0.86	0.62	0.51	0.72	0.02	0.69	0.29	0.59
RF	0.99	1	0.8	0.7	0.88	0	0.69	0.71	0.71
LASSO	0.7	0.62	0.57	0.46	0.68	0.12	1	0	0.7
ANN	0.92	0.89	0.71	0.6	0.8	0	0.5	0.64	0.55

*Abbreviations*: *RF* Random forest, *ANN* Artificial neural network, *ACC* Accuracy, *tr* training, *tst* Test, *p* states for p(ACC > no information rate): it is the probability (*p*) of the accuracy (*ACC*) being greater than the no information rate. F1 score is a combined metric that considers both precision and recall, providing a balanced measure of a model’s performance. L and U are 95% confidence intervals of accuracy

**Fig. 6:**
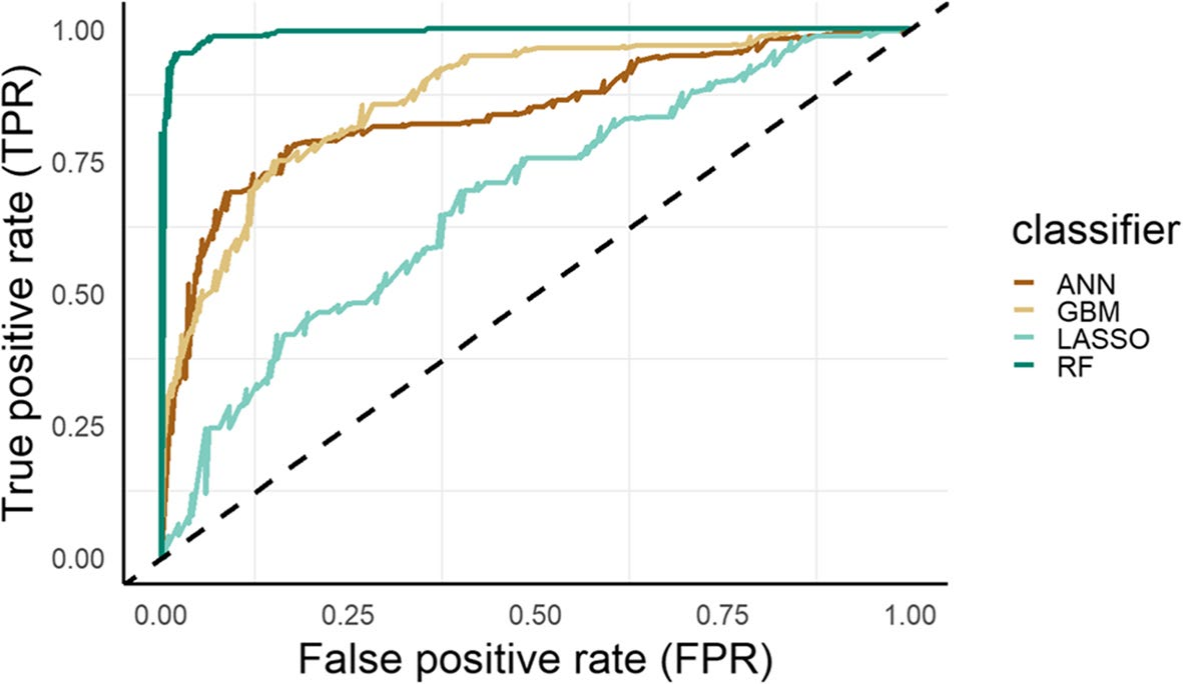
The area under the receiver operating characteristic (ROC) curve (AUC) of the considered models. False-positive rate (FPR) and true-negative rate (TPR) were considered. The plot shows the ROC curves calculated for each classifier over the entire dataset. RF offers the best performance. Abbreviations: LASSO, LASSO–RIDGE regression; GBM, gradient boosting machine; ANN, artificial neural network; RF, random forest

The GBM demonstrated higher sensitivity, correctly identifying over two-thirds of cancer patients who required multiple teleconsultations (sensitivity = 0.69). In terms of specificity, RF achieved the highest score among the models, followed by ANN (specificity = 0.71 for RF and 0.64 for ANN). On the other hand, LASSO performed poorly, incorrectly predicting that all individuals would require multiple remote consultations (Fig. 6).

### Simulated risk analysis

The best ML model (i.e., RF) was adopted to perform simulated odds ratios (sORs) of different cancer patients’ profiles [8]. Each individual was simulated 200 times to predict the risk percentage of doing more than one remote consultation, and such risk was replicated 300 times. Therefore, a 300-large sample of target cancer patients and reference cancer patients were created as odds and discriminant conditions were compared as odds ratio measures. Conditions were left as fixed data, while other features were randomly chosen. In particular, the site of the tumor was treated as a gender-dependent feature, to build plausible profiles.

**Fig. 7:**
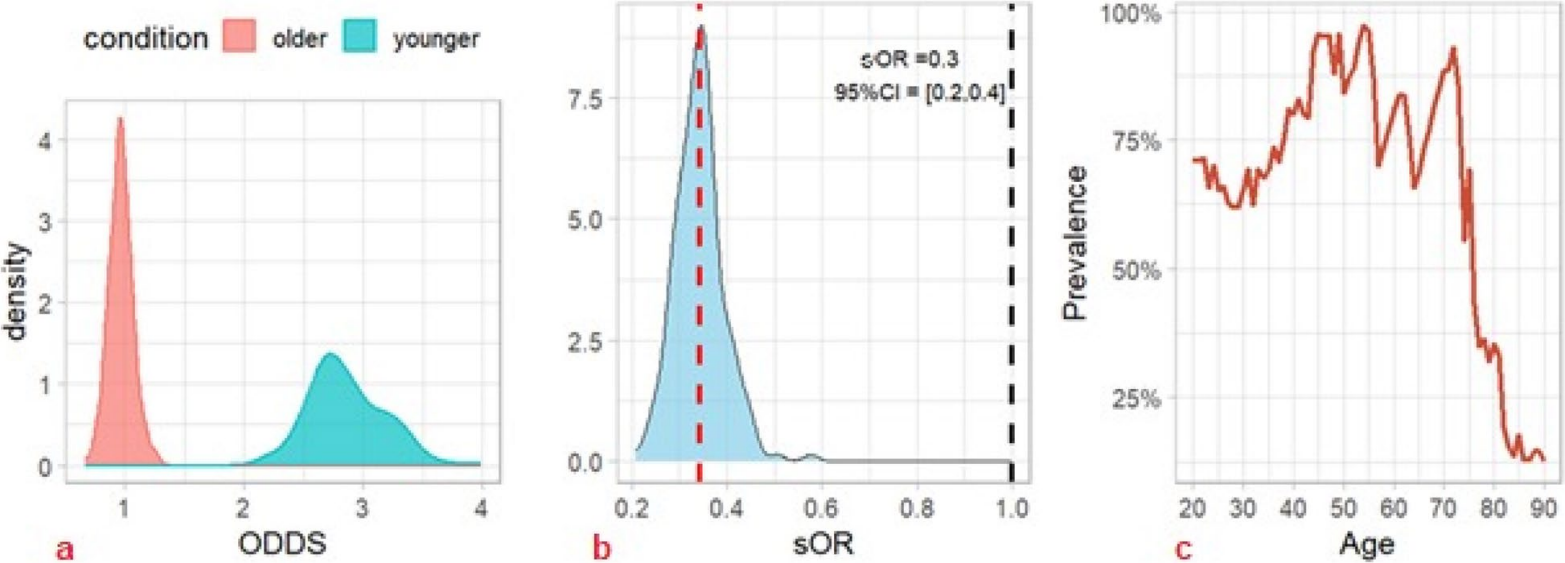
Machine learning simulation on age. We simulated two cohorts of 300 cancer patients having a probabilistically Gaussian age: younger (45 years old) vs. older (75 years old) (*SD* = 5). The remaining features of the dataset were randomly generated and distributed uniformly across all cohorts. Older cancer patients had a significantly lower risk (− 70%) of receiving multiple remote consultations (*sOR* = 0.3, 95% *CI* = [0.2–0.4]) (red dashed line). This risk reduction remained relatively constant until the age of 75 and then rapidly decreased to zero (Fig. 7c). Abbreviations: sOR, simulated odds ratio; ODDS, odd ratios. The black dashed line symbolizes an sOR of 1, indicating a condition of no association

**Fig. 8:**
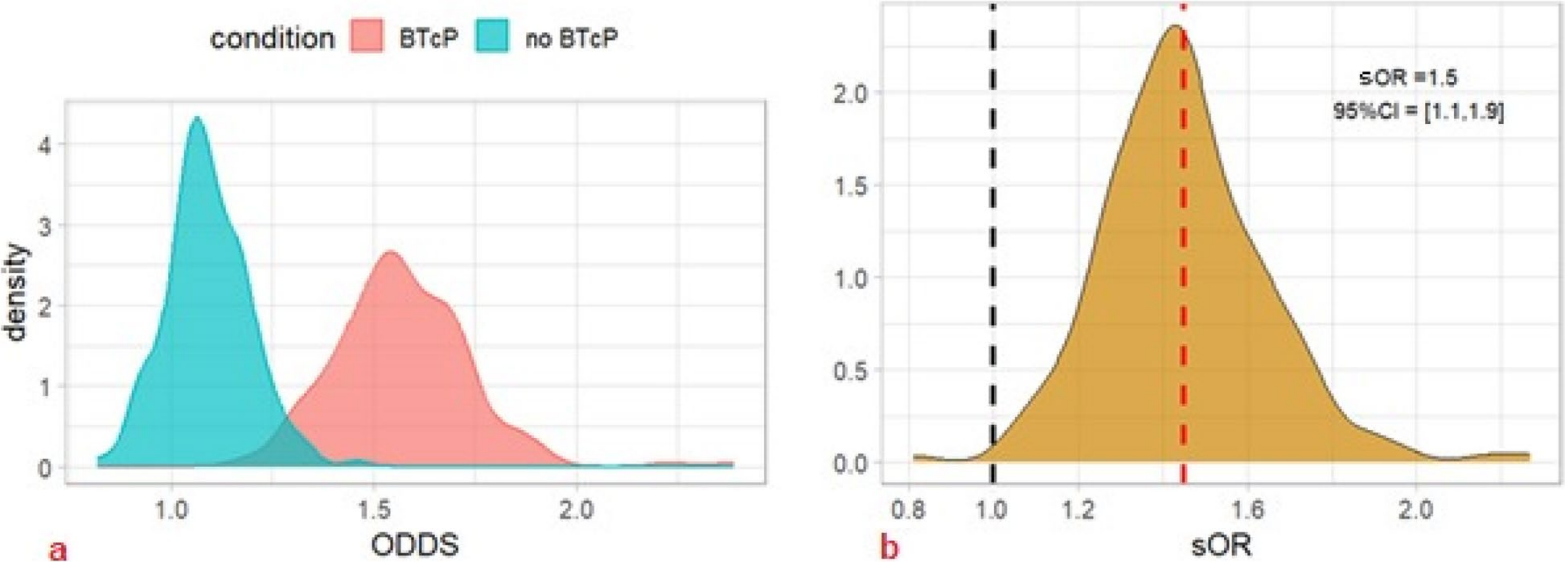
Machine learning simulation on breakthrough cancer pain. We simulated two cohorts of 300 cancer patients affected or not by BTcP. The simulation analysis revealed a significantly higher risk for cancer patients who experienced breakthrough cancer pain (BTcP) to undergo more than one remote consultation (*sOR* = 1.5, 95% *CI* = [1.1, 1.9]) (red dashed line). Abbreviations: sOR, simulated odds ratio; ODDS, odd ratios. The black dashed line symbolizes an sOR of 1, indicating a condition of no association

**Fig. 9:**
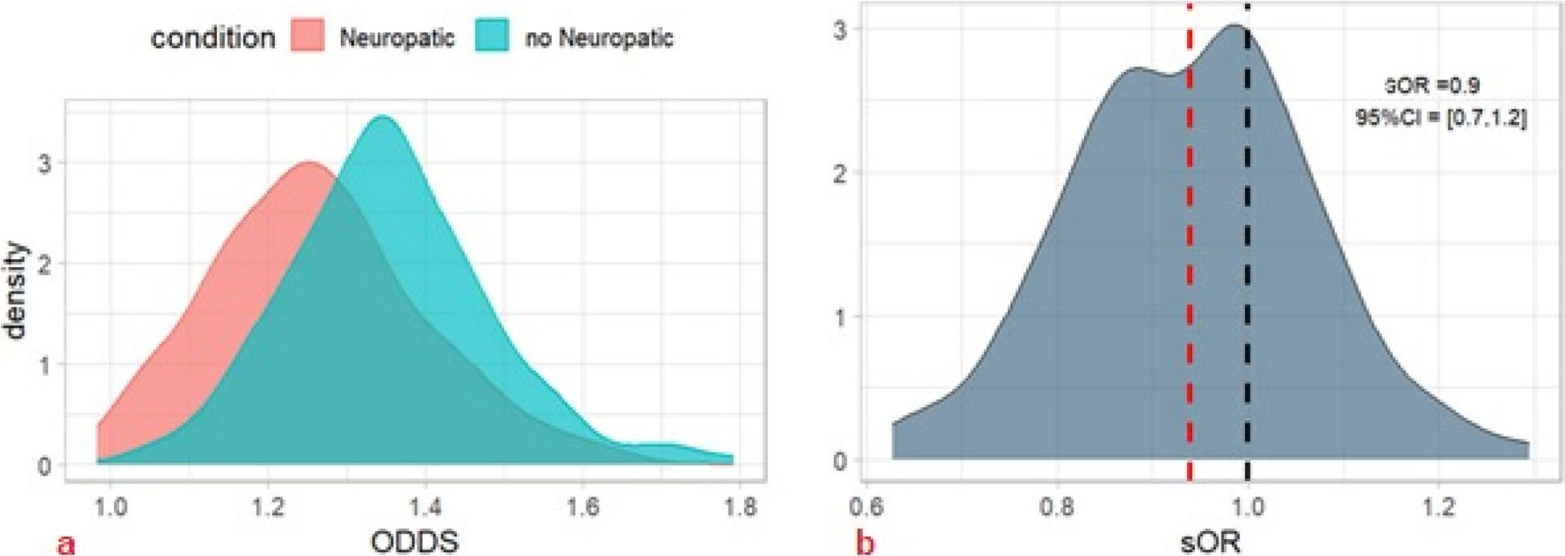
Machine learning simulation neuropathic pain. Regarding the neuropathic pain condition, our analysis did not observe a significant difference between the two profiles, with a mean simulated odds ratio (sOR) of 0.9 (95% *CI* = [0.7, 1.2]) (red dashed line)

• Condition 1: A comparison was made between a young cancer patient and an older one, considering age as a Gaussian distribution with a standard deviation of 5. Younger individuals had a mean age of 45 years old, while older individuals had a mean age of 75 years old. The other features were kept as randomly chosen. Older cancer patients had a − 70% risk of receiving multiple remote consultations (*sOR* = 0.3, 95% *CI* = [0.2–0.4]); see Fig. 7 a and b. In particular, the risk was almost constant until 75 years of age and then decreased rapidly to 0 (Fig. 7c).
• Condition 2: Concerning the variable BTcP, the simulation proved a significantly higher risk for more than one remote consultation for cancer patients who suffered from this cancer pain phenomenon (*sOR* = 1.5, 95% *CI* = [1.1, 1.9]) (Fig. 8).
• Condition 3: Concerning neuropathic pain, our results did not find a significant difference between the two profiles. The mean sOR was 0.9 (95% *CI* = [0.7, 1.2]) (Fig. 9). At the same time, the double condition of BTcP and neuropathic pain was not a risk factor for a higher risk of more teleconsultations (*sOR* = 1.1, 95% *CI* = [0.8,1.5], not shown).

## Discussion

There is a significant degree of uncertainty surrounding the appropriate care model for managing cancer pain through telemedicine. With a lack of established guidelines and limited data from existing literature, we have previously developed a “hybrid” approach that combines both in-person and remote components [6]. By adopting this comprehensive and adaptable approach, we aim to optimize the quality of care delivered to patients experiencing cancer pain while utilizing the benefits of telemedicine [7].

Therefore, to improve the pathway, we have implemented an AI technique. AI can facilitate the integration of telemedicine into clinical practice by streamlining various processes. For example, the implementation of AI-driven triage systems can effectively prioritize patient cases according to their severity, guaranteeing that those requiring immediate attention receive prompt care. Moreover, AI algorithms can aid in the analysis of diagnostic images, facilitating precise and efficient remote diagnoses. This not only saves valuable time but also guarantees that patients receive timely and accurate assessments, resulting in enhanced outcomes [22].

GANs have emerged as a powerful tool in medical research, offering new opportunities and advancements in various areas of healthcare. These strategies have been employed for data synthesis, enabling the generation of synthetic medical data that can be used to augment real-world datasets and overcome limitations in data availability [10]. In the context of our study, the generated dataset had a distribution that closely aligned with the reference dataset across various variables. The similarities between the generated and the reference dataset imply that the synthetic data accurately captures the patterns and characteristics observed in real-world data. Consequently, by successfully replicating the distribution of these variables, the generated dataset becomes a valuable resource for training and evaluating ML models. These factors reinforce the utility and validity of GAN-based datasets for various applications, such as predictive modeling, decision support systems, and clinical research, especially when real data may be limited or restricted due to privacy concerns or data availability [23].

Within the framework of our telemedicine clinical practice, we noticed that while many cancer patients required a single consultation, there were individuals who needed a higher number of closely spaced remote visits [5, 13]. This evidence prompted us to investigate the characteristics of cancer patients who may necessitate more than one remote consultation to ultimately develop personalized care pathways and optimize resource allocation. Therefore, to achieve internal and external validation and implement the chosen model in clinical settings, we explored various ML models. We opted to categorize the number of remote consultations as either “one” or “more than one” and utilized this classification for prediction purposes.

The simulation analysis can provide a comprehensive examination of ML methods by enhancing the dataset and emphasizing distinctive data point distributions. This approach can offer ample opportunities for in-depth exploration and study, benefiting from the remarkable capabilities of nonparametric methods to uncover hidden patterns within the data [24]. In our study, the simulated risk analysis demonstrated that the risk of having more tele-visits decreased with age. Moreover, individuals with BTcP have an increased likelihood (approximately 50% more) of requiring multiple remote consultations compared to those without this condition. These data reaffirm our previous findings from an analysis specifically focused on the phenomenon of BTcP. In a hierarchical classification, we observed that the most severe phenotype of cancer pain patients was characterized by the presence of BTcP in conjunction with younger age [25]. This combination of factors identified a subgroup of patients experiencing a more challenging pain profile, highlighting the need for targeted interventions and comprehensive pain management strategies for this specific patient population [26].

In contrast, when examining the variable of neuropathic pain, our analysis did not uncover a significant difference between individuals affected by neuropathic pain and those who were not. Additionally, the combination of BTcP and neuropathic pain did not demonstrate an association with an increased risk of requiring a greater number of teleconsultations. These findings suggest that, in terms of teleconsultation frequency, the presence of neuropathic pain alone or in conjunction with BTcP may not significantly impact the clinical management of cancer patients. Probably, these factors contribute to an increased demand for in-person visits [5]. These data call for further investigation. With the progressive implementation of the dataset, we will certainly be able to conduct more in-depth statistical and predictive analyses. At this stage, we can only speculate that the presence of neuropathic pain associated with oncological conditions identifies a subgroup of particularly complex patients with a need for frequent hospital visits. For example, in a previous analysis, we observed a correlation between the number of visits and pharmacological therapies for neuropathic pain and the risk of hospital readmission [5].

### Limitations

Implementing GANs in medical research presents challenges alongside their potential benefits. These challenges concern data quality and interpretability of the generated outputs, while ethical considerations play a crucial role in ensuring the reliability and responsible use of GAN-generated results. Data quality is a fundamental concern as GANs require large, high-quality datasets for effective training. However, medical datasets often suffer from limitations such as missing data, imbalances, and potential errors, which can affect the accuracy and reliability of GAN outputs. Interpretability is another issue surrounding GANs. The generated results may lack clear explanations or rationales for the decisions made by the model. In medical applications, where transparency and understanding are vital, this lack of interpretability can hinder trust and adoption.

Ethical considerations are paramount in using GAN results in medical research. These considerations encompass patient privacy, informed consent, and the risk of unintended biases. Stringent privacy regulations must be followed to protect patient confidentiality when using medical data in GANs. Obtaining informed consent from patients whose data is used for training is essential. Additionally, there is a potential for unintended biases to be encoded in the generated outputs, leading to disparities or unfairness in healthcare decision-making [27]. Therefore, establishing robust frameworks and guidelines becomes imperative to govern the implementation of GANs, safeguard patient privacy, mitigate biases, and promote transparency and accountability throughout the development and deployment of these models [28, 29].

Furthermore, training GANs can be a challenging task that demands meticulous parameter tuning. Additionally, GANs are susceptible to mode collapse, a phenomenon where the generator fails to explore the complete spectrum of possible data and instead produces limited variations. Researchers are actively investigating and enhancing GAN architectures and training methodologies to address these limitations and unleash the complete potential of these AI strategies [30].

A significant limitation is the limited availability of samples, which could potentially undermine the reliability of the analysis and increase the risk of encountering issues such as overfitting, where the model becomes too closely tailored to the available data and may not generalize well to new or unseen cases [31]. It is crucial to acknowledge this limitation and exercise caution when interpreting the results while also considering strategies to mitigate these challenges, such as obtaining larger and more diverse datasets or employing advanced regularization techniques during the model training process. On the other hand, it would be beneficial to contemplate the option of augmenting the dataset to encompass additional variables such as long-term outcomes, patient satisfaction, and quality of life. Furthermore, including data from other institutions that have implemented the same telemedicine pathway could provide a broader perspective and further strengthen the findings of the study.

Finally, concerning model evaluation, we conducted a small number of simulations to display the model’s application. However, it is crucial to highlight that the evaluated model has the capacity to be applied across a vast array of variable combinations. Therefore, we are pleased to offer the dataset and model for further investigation upon request.

## Conclusion

As telemedicine continues to advance and gain widespread acceptance, it is imperative to conduct thorough research and evaluation to fully uncover its immense potential in managing pain associated with cancer. By harnessing the power of telemedicine, healthcare professionals can improve access to care, enhance patient outcomes, and provide comprehensive pain management services. On the other hand, the refinement of the healthcare process requires scientific evidence. Therefore, the use of AI techniques can help bridge knowledge gaps and accelerate the integration of telemedicine into clinical practice, providing more personalized and effective care and ultimately improving patient outcomes. However, it is crucial to approach the utilization of GANs and other AI frameworks in medical research with careful consideration, transparency, and a comprehensive understanding of their capabilities and limitations. Lastly, although the study primarily focused on evaluating the performance of various ML algorithms, it would be beneficial to enhance it by comparing the AI framework’s performance with standard clinical approaches.

## Data Availability

The datasets used and/or analyzed during the current study are available at [13] and [21].
